# Announcement

**Published:** 2015-03-27

## Autism Awareness Month and World Autism Day — April 2015

April is Autism Awareness Month, and April 2 is World Autism Day. These observances offer the opportunity to highlight the increasing number of children identified with autism spectrum disorder (ASD) and the substantial burden on families and health, educational and other support services, as well as an opportunity to celebrate the unique perspectives of those living with ASD.

ASD is a developmental disability that can cause major social, communication, and behavioral challenges. Signs of ASD begin during early childhood and usually last throughout a person’s life (1). The cause of most cases of ASD is unknown, and there is currently no cure. CDC’s most recent surveillance data indicate that about one in 68 children has been identified with ASD (2), which represents an almost 30% increase since the previous estimate in 2012. CDC has been active in documenting changes in the number and characteristics of children with ASD over the past decade. However, there remains an urgent need to continue research into causes of and effective interventions for ASD (3) and help children living with ASD to achieve their potential.

CDC, working with its state and academic partners, is committed to tracking the changing number and characteristics of children with ASD, researching what puts children at greater risk for ASD, and promoting early identification of children with ASD.

Information about CDC’s data on ASD is available at http://www.cdc.gov/ADDM. Information on CDC’s study for understanding risk factors and causes of ASD is available at http://www.cdc.gov/SEED. Resources to help parents, health care providers, and early childhood care and education providers track each child’s development are available for download free of charge at http://www.cdc.gov/ActEarly.
